# “Frenemies” of innovation: understanding the role of coopetition in service innovation in emerging markets

**DOI:** 10.12688/openreseurope.14472.2

**Published:** 2022-06-21

**Authors:** Sohaib S. Hassan, Abiodun Egbetokun, Levan Bzhalava

**Affiliations:** 1 BMBF-KontiKat, Faculty III, University of Siegen, Siegen, NRW, 57072, Germany; 2 National Centre for Technology Management, Obafemi Awolowo University, Ile-Ife, 220005, Nigeria; 3 Finland Futures Research Centre, Turku School of Economics, University of Turku, Turku, FI-20014, Finland

**Keywords:** Coopetition, Emerging Markets, Innovation, SMEs

## Abstract

Coopetition is considered an important strategy for innovation. However, the literature provides limited evidence on how coopetition relates to innovation in service sector, particularly in emerging markets. Moreover, little is known about the effects of the formal and informal aspects of coopetition on innovation and how absorptive capacity of firm may influence this relationship. Against this background, using the official national innovation surveys of Nigeria (2008 and 2011), this study contributes to the ongoing debate by empirically examining the innovation endeavors of 421 Nigerian SMEs. The study employs logistic regression methods to model and explore the relationships between coopetition and innovation in the sample. The results show that that formal coopetition hinders innovation while informal coopetition supports it and absorptive capacity moderates these relationships. The study provides important insights about the concept of coopetition in emerging markets, especially vis-à-vis their institutional idiosyncrasies. Finally, the study highlights its implications and suggests some avenues for future research.

## 2. Plain language summary

Firms innovate by collaborating within their networks and with partners. Sometimes though firms cooperate with their competitors to generate innovation, *e.g*., as in the case of open-source software and technologies. Such type of innovation is known as coopetition. Further, collaboration in coopetition could be through official channels (formal, *e.g*., contracts, agreements) or unofficial channels (informal, *e.g*., social gatherings). The literature has already shown how large companies in the advanced economies of the developed world use coopetition to increase their innovation output. However, we do not know much about coopetition among small firms of the developing/emerging markets. This study addresses this issue and carries out an empirical analysis in this regard. We use a survey of small and medium enterprises in Nigeria to see how innovation there is influenced by the official and unofficial channels of coopetition. Our results show that that formal coopetition hinders innovation while informal coopetition supports it and absorptive capacity (a firm’s ability to learn) moderates these relationships. We also discuss the implications and limitations of our study.

## 3. Introduction

Firms tend to collaborate externally due to the costs involved in internal knowledge creation and the locus of innovation being in networks rather than in individual firms ( Hagedoorn, 1993; Powell *et al.*, 1996). The dynamics of inter-firm collaboration have long been viewed from the lens of business partnerships and alliances. The management research underscores that firms do not only cooperate with partners in a network, but also with rivals, the so-called “coopetition” ( Dagnino & Padula, 2002; Das & Tang, 2000; Nalebuff & Brandenburger, 1997).

The strategic management literature consistently suggests that coopetition can be good for innovation ( Quintana-García & Benavides-Velasco, 2004; Ritala, 2012; Tether, 2002), as competitors or “frenemies” ^1^ operate in similar parameters ( Bouncken & Kraus, 2013; Enberg, 2012). However, it is not yet clear how coopetition interplays with the prospects of innovation, particularly in relation to the different types of coopetitive interactions and the contextual factors that influence coopetition ( Bengtsson & Kock, 2014). Existing research suggests that coopetition between firms can either be through formal channels ( Brusoni *et al.*, 2001; Hagedoorn *et al.*, 2000; Tether, 2002) or informal channels ( Freeman, 1991; Tödtling *et al.*, 2009). Further, the literature underscores that a firm’s absorptive capacity, similar to other collaboration processes, may define the extent of benefits a firm can gain through formal or informal coopetition ( Cohen & Levinthal, 1990; Ritala & Hurmelinna-Laukkanen, 2013; Zahra & George, 2002). Taken together, this highlights a gap in the literature and leads us to our main research question: how do formal and informal coopetition relate to innovation, and how does absorptive capacity moderate this relationship?

On one hand, much of the empirical literature on innovation concentrates on technological product and process innovation, with much less emphasis on service innovation ( Adeyeye *et al.*, 2013). Service innovations, particularly in emerging countries, are driven by different needs than that of advanced economies ( Phills *et al.*, 2008). On the other hand, the existing literature about coopetition focuses largely on understanding the influence of coopetition on innovation in high-technology sectors and knowledge intensive industries of advanced economies (e.g., Arranz & de Arroyabe, 2008; Hagedoorn & Schakenraad, 1992; Nieto & Santamaría, 2007), whereas the research about small and medium enterprises (SMEs) in emerging economies is rather limited ( Bengtsson & Johansson, 2014). SMEs tend to differ from large firms in terms of their competences and priorities. Further, due to their rather unsystematic structures, SMEs show relatively less interest in pursuing coopetition strategies to gain performance outcomes ( Morris *et al.*, 2007; Roig-Tierno *et al.*, 2018). However, while SMEs are generally market oriented and tend to mainly focus on growth and developing their core businesses, they need to be innovative in competitive markets to overcome their liabilities of smallness ( Bengtsson & Johansson, 2014; Roig-Tierno *et al.*, 2018). Coopetitive alliances might facilitate their competitive advantage, access to new markets and survival ( Kraus *et al.*, 2012; Morris *et al.*, 2007). The existing research indeed has pointed to such practices among SMEs (e.g., Bouncken *et al.*, 2015; Crick & Crick, 2021; Devece *et al.*, 2019; Gernsheimer *et al.*, 2021; Kraus *et al.*, 2012), but the empirical evidence is rather limited in scope (sectoral and geographical).

Further, the existing research in this regard deals mostly either with the multinational organizations’ alliances with the local partners or governments in emerging markets ( Kedia *et al.*, 2016) or with the performance related aspects of coopetition ( Shen *et al.*, 2019). Thus, little research is available on the relationship between coopetition and innovation among the local SMEs. There is indeed an increasing debate about how contextual factors and institutional aspects might influence coopetition ( Barney *et al.*, 2016; Dagnino *et al.*, 2012). For instance, innovation in emerging markets is often idiosyncratic and closely connected to the social fabric of their respective societies. The results obtained from other contexts may therefore not be fully applicable to policy and practice in emerging contexts. By providing fresh empirical results on the service innovation of SMEs in an emerging economy, this paper makes valuable contributions to the innovation management literature. Our panel dataset of 421 service industry SMEs comes from Nigeria’s official innovation surveys of 2008 and 2011. The rest of this study proceeds as follows: we review the theoretical development and establish our hypotheses in the next section. In Section 3, we introduce the data used and the methodology adopted for this study. We present and discuss our empirical findings in Section 4 and we conclude in Section 5.

## 4. Theoretical background and hypotheses

Innovation is one of the key components of a firm’s strategy and is central to economic competition ( Schumpeter, 1934). In the extant scholarship, the resource-based view of the firm underscores that the firms gain competitive advantage through a combination (bundle) of generic, internal resources and production capabilities ( Barney, 1991; Wernerfelt, 1995). Internal resources, both tangible and intangible ones are central and often unique to a firm. The exploitable nature of such resources, coupled with the aspects of organizational learning, determine the competitive positioning of a firm in imperfect markets ( Barney *et al.*, 2011; Kogut & Zander, 1996). The knowledge-based view of the firm complements the notion of resources by identifying the strategic and central role of knowledge in shaping the competitive position of a firm through innovation and inimitable internal resources ( DeCarolis, 2002; Grant, 1997; Grant, 2002; Spender & Grant, 1996; Subramaniam & Youndt, 2005).

However, contrary to physical resources, the knowledge required for innovation is costly to create internally and its rather intangible nature makes it very difficult to acquire through market transactions ( Shelanski & Klein, 1995). Firms then have the option of accessing external knowledge assets to complement their internal capabilities through cooperation with other firms ( Brusoni *et al.*, 2001). While, cooperation between firms is traditional and logically entails the collaboration between partners in achieving similar objectives, scholars in management research have pointed out the potential of cooperation between competing firms (henceforth, coopetition) for innovation ( Bengtsson & Kock, 1999; Bengtsson & Kock, 2014; Brandenburger & Nalebuff, 1996; Tether, 2002).

A number of studies have demonstrated that firms, instead of working alone, tend to engage in inter-firm cooperation with their competitors to create value and resources ( Bengtsson & Kock, 2014; Tether, 2002; Zineldin, 2004). Similarly, network theory accentuates that cooperation with competitors does not only provide firms with the opportunities to learn about and from the competitors, but also enables them to benefit from the pool of collective resources within a network ( Lado *et al.*, 1992; Lado *et al.*, 1997). The underlying logic is that competitors work within similar technological and knowledge paradigms as well as along similar trajectories leading them to cooperate and compete with each other at the same time ( Bengtsson & Kock, 2000; Brandenburger & Nalebuff, 1996). Thus, they tend to hold similar fundamental knowledge endowments; and similarity in their knowledge base is conducive to knowledge sharing and knowledge integration ( Bouncken & Kraus, 2013).

Competitors in partnerships are believed to benefit from the synergies created through the collaboration among firms which complement each other’s resources and capabilities, and thus generate innovation ( Brandenburger & Nalebuff, 1996; Carayannis & Alexander, 1999; Quintana-García & Benavides-Velasco, 2004; Tether, 2002). However, research in evolutionary economics, while broadly agreeing with the above ( Gnyawali & Park, 2009; Nelson, 1990), raises concerns about the benefits of coopetition. In particular, the innovation potential of cooperation is shown to reduce with similarity in the knowledge bases of the partners. More specifically, the greater the cognitive proximity between partners, the more they tend to understand each other and share knowledge but the less the potential of their cooperation to generate novelty. A trade-off therefore arises between technological/cognitive overlap and technological/cognitive distance ( Egbetokun & Savin, 2015; Gilsing *et al.*, 2008; Mowery *et al.*, 1998; Nooteboom *et al.*, 2007; Wuyts *et al.*, 2005).

### 2.1 Coopetition channels, innovation and SMEs

The literature identifies two channels through which coopetition can take place: formal and informal ( Brusoni *et al.*, 2001). Formal coopetition, like any other official inter-firm collaboration, consists of legally binding commitments and contracts between the signatories. Such contracts are focused on certain procedures through which competitors make an alliance or partnership to pursue specific objectives, such as technology-sharing agreements, joint research initiatives or product development ( Hagedoorn, 2002; Tether, 2002). Informal coopetition consists of information exchange through unofficial networking events, such as in meetings, exhibitions and conferences ( Pyka, 1997; Tödtling *et al.*, 2009). The existing research underscores that while both the channels lead to positive innovation outcomes, informal coopetition tend to be more frequent due to their convenient, cost-effective less explicit nature ( Egbetokun, 2015; Pyka, 1997).

Coopetition contains certain inherent disadvantages such as imitation risk, unintentional knowledge spill-overs and inconducive trust-deficit between the participating agents ( Bouncken & Kraus, 2013; Jaffe *et al.*, 1993; Phene & Tallman, 2014; Ritala & Hurmelinna-Laukkanen, 2013). In formal coopetition, firms employ different legal appropriability mechanisms (e.g., patents, trademarks etc.) to protect their core competences from their rivals and to reduce the risks of imitation ( Somaya, 2003). Similarly, formal coopetition is associated with a delicate balance of reciprocal knowledge transfer, *i.e.*, between acquiring new knowledge, while at the same time, safeguarding firms’ own knowledge resources ( Fehr & Gächter, 2000; Hamel *et al.*, 1989b; Kogut & Zander, 1996; Levy *et al.*, 2003; Loebecke *et al.*, 1999). When it comes to coopetition for innovation, the risks of unintentional knowledge leakage, opportunism and imitation are much higher ( Hamel *et al.*, 1989a; Kogut & Zander, 1996; Loebecke *et al.*, 1999; Ritala & Hurmelinna-Laukkanen, 2009). Larger firms can act proactively against the unintended leakage of information to their competitors and establish agreements that entail strict mechanisms of knowledge sharing; however, strict appropriability mechanisms may limit the willingness of smaller firms to collaborate with larger partners ( Liebeskind, 1997). However, such reciprocity-related risks may be absent in informal cooperation even when the partner is a competitor.

Two factors are relevant in explaining the choice of firms between formal and informal coopetition. First, the size of organization matters, as the larger organizations, because of their resources, can offset the costs and risks involved in formal knowledge sharing mechanisms with their competitors in regional and international markets ( Luo, 2005). The literature on the dynamics of inter-firm collaboration focuses mainly on large organizations as their international relocation of resources often requires them to be part of alliances and partnerships with local partners and competitors to mitigate their foreignness ( Akdoğan & Cingšz, 2012; Kedia *et al.*, 2016; Luo, 2007). Second, the quality of institutions also plays an important role. Formal coopetition thrives in countries with better institution and legal infrastructure, where formal agreements are overlooked and supported by the responsible agencies ( Kylänen & Rusko, 2011). Consequently, much of the existing research focuses on large organizations and multinationals from the advanced countries and high-tech manufacturing, while the focus on SMEs and the service sector is rather unexplored ( Luo, 2005; Thomason *et al.*, 2013), although as highlighted earlier, coopetition indeed offers some benefits to SMEs too ( Bengtsson & Johansson, 2014).

Building upon the foregoing, we argue that formal coopetition agreements can be costly in emerging markets because of weak institutions. SMEs in emerging markets are more exposed to institutional deficits because of their limited resources, liability of smallness as well as their strong focus on traditional industrial activities, and this leads them to prefer informal interactions where mutual trust and commitments substitute the institutional deficiencies ( Biggs & Shah, 2006; Oyelaran-Oyeyinka & Banji, 2006). Further, the social networks in the emerging economies should be conducive to informal exchange of information for innovation activities between the competitors because the inherent element of trust *vis-à-vis* the informal networks favors complementary knowledge sharing ( Murphy, 2002; Thomason *et al.*, 2013).

This discussion leads to our first set of hypotheses:

- **Hypothesis 1a:** *Formal coopetition is negatively associated with the probability to innovate in SMEs in an emerging economy*
- **Hypothesis 1b:** *Informal coopetition is positively associated with the probability to innovate in SMEs in an emerging economy*

### 2.2 Moderating role of absorptive capacity

A firm could either benefit or lose from inter-firm collaboration and coopetition, and this can be affected by the internal organizational structure of the firm ( Foss *et al.*, 2013; Ritala & Hurmelinna-Laukkanen, 2013). One way by which firms manage the coopetition cost–benefit trade-off is to accumulate absorptive capacity which enhances knowledge search, valuation, assimilation and appropriation ( Cohen & Levinthal, 1990; Zahra & George, 2002). The concept of absorptive capacity is rooted in the individual cognitive dimensions for problem solving, learning new competences to generate new ideas and cumulative learning of firms ( Cohen & Levinthal, 1989; Cohen & Levinthal, 1990). This capacity, for instance, can be developed through investments in R&D ( Cohen & Levinthal, 1989) and human capital ( Cohen & Levinthal, 1994). As indicated by recent research, absorptive capacity indeed widens and lengthens the reach of collaboration such that firms can collaborate with distant partners ( Berchicci *et al.*, 2016; de Jong & Freel, 2010; Drejer & Vinding, 2007) and even internationally ( Ebersberger & Herstad, 2013).

 The absorptive capacities of firms vary according to their previous knowledge stock and capacities to identify and assimilate knowledge flows ( Cohen & Levinthal, 1989; Cohen & Levinthal, 1990; King & Lakhani, 2011; Quintana-García & Benavides-Velasco, 2004). When firms cooperate with each other, the diffusion of knowledge across individuals’ and firms’ boundaries is considered essential for the development of innovation capacities and the profit maximizations of firms ( Grossman & Helpman, 1991). Similarly, since firms in coopetition alliances often share a common knowledge base and cognitive proximities relative to each other, they are able to communicate efficiently and increase their absorptive capacities over time ( Knoben & Oerlemans, 2006). However, the extent to which firms will benefit from the knowledge exchange to innovate depends on their respective absorptive capacities. Firms with higher absorptive capacities are better off in building on their existing competences within an alliance, relative to the firms with lower levels of absorptive capacities. ( Boschma, 2005; Cohen & Levinthal, 1994; Dussauge *et al.*, 2000; Ritala & Hurmelinna-Laukkanen, 2013). Moreover, firms with higher level of previous knowledge accumulation, experience and greater capacities to assimilate new knowledge make their employee better informed, more innovation oriented, and increasingly capable of knowledge and information assimilation, compared to the firms with lower levels of absorptive capacities ( García-Morales *et al.*, 2012).

Firms competing and cooperating at the same time are prone to the risk of opportunism on the part of their competitors ( Brandenburger & Nalebuff, 1996; Quintana-García & Benavides-Velasco, 2004; Tether, 2002). One way to mitigate this risk is to formalize the coopetition alliance. In a formal coopetition partnership, firms with higher absorptive capacities are better prepared to counter the risk of opportunism due to their higher level of absorptive capacities. Moreover, the absorptive capacities are believed to influence the coopetition management between firms. Firms with higher absorptive capacities are in a better position to strategically manage their knowledge sharing mechanisms and set out the rules of the game and prevent their competitors against unnecessary opportunism and have a chance to learn from their competitors, even opportunistically ( Levy *et al.*, 2003). This debate leads to our second set of hypotheses:

- **Hypothesis 2a:** *Absorptive capacity positively moderates the association between formal coopetition and innovation*
- **Hypothesis 2b:** *Absorptive capacity negatively moderates the association between informal coopetition and innovation*

## 5. Methods

### Data and sample

Our data comes from the two waves of Nigeria’s official national innovation surveys that are available: the first wave covering 2005–2007 was completed in 2008 and the second wave covering 2008–2010 was completed in 2011. The surveys are based on the Oslo Manual and, hence, share the core set of questions with the Community Innovation Surveys (CIS) of Europe. The datasets, which are openly available online ( https:/doi.org/10.17632/37pys4vxt4.1), include information on the innovation investments, sources, obstacles, and outcomes in the firms as well as detailed firm characteristics including size, human capital, age, location and export status. The datasets have been widely applied in recent research ( Edeh & Acedo, 2021; Medase, 2020; Medase & Wyrwich, 2021) which provide more specific details on the survey methodology.

The two waves of the survey represent two repeated cross sections of firms selected by stratified random sampling across the manufacturing and services sectors at the two-digit ISIC level (see A. Egbetokun, 2017). Although it was ensured that every firm that responded in the first wave was contacted for the second wave, the response was particularly low, necessitating a re-sampling following the procedure just described. Consequently, the final sample size across both waves of the survey is not the same and only about 2.5% of the firms appear in both cross sections. For this reason, we are unable to perform a longitudinal analysis. Nonetheless, the amount of information contained in the datasets and their comparability with data from other countries make them very useful for rigorous empirical analyses. The full dataset includes 1359 firms from both waves of the survey, of which 469 (34.5%) are from the services sector. Our final sample includes 421 firms (179 firms in the first wave and 242 in the second wave) because the remaining 48 did not perform any innovation and thus, were not eligible to respond to the cooperation and information sources questions in the survey. A more detailed sectoral breakdown of the sample, using two-digit ISIC classification, is presented in Table 1.

**Table 1.** Sectoral distribution of sample.

Two-digit ISIC sector	Number of firms	Percentage of sample
Health, cultural and social work	117	27.79
Trade, repairs, and rentals	61	14.49
Other business activities	46	10.93
Hotels and restaurants	40	9.50
Transport services	40	9.50
Computer and related activities	35	8.31
Insurance and pension funding	26	6.18
Financial intermediation	20	4.75
Real estate activities	16	3.80
Post and telecommunications	11	2.61
Education	9	2.14
Total	421	100.00

### Variables and descriptive statistics

***Dependent variables*.** To understand the influence of coopetition and absorptive capacity on innovation, we use two binary measures of *Product innovation* and *Process innovation* as our dependent variables. The variables contain the information whether a firm introduced an innovation (product, process) during the reference period. This information is the best available in the survey and it is the standard way of measuring innovation in CIS-type innovation surveys.

***Independent variables*.** We use a binary measure for our explanatory variable of *Formal coopetition*, that is, whether a firm collaborated with a competitor during the reference period or not. Similarly, we use a binary measure for our explanatory variable of *Informal coopetition*. Further, we use *Staff quality* and *Staff training* as the proxies for absorptive capacity of a firm, that is, a firm’s ability to benefit from the inter-firm collaboration.

***Control variables*.** We also use several control variables, traditionally associated with firms’ collaboration for innovation. A detailed list of names, description and measurements of variables is provided in Table 2.

**Table 2.** Description of variables.

Variable	Description	Measure
*Dependent variables*		
Product innovation	Binary measure of whether a firm introduced a new product between 2005–2007 or 2008–2010	=1 if Yes; 0 if No
Process innovation	Binary measure of whether a firm introduced a new product between 2005–2007 or 2008–2010	=1 if Yes; 0 if No
*Independent variables*		
Formal coopetition	Binary measure of whether a firm collaborated with a competitor during the reference period	=1 if Yes; 0 if No
Informal coopetition	Binary measure of whether a firm used a competitor as source of innovation information during the reference period	=1 if Yes; 0 if No
Staff quality	Percent of staff with at least a bachelor’s degree	Continuous between 0 and 1
Staff training	Binary measure of whether the firm officially organizes periodic staff training	=1 if Yes; 0 if No
*Controls*		
Other collaboration partners	Binary indicator of whether the firm had any other partner during the reference period apart from competitors	=1 if Yes; 0 if No
Size	Number of employees	Continuous
Age	Number of years since establishment of the firm	Continuous
Location	Binary measure of whether the firm is located in Lagos, the major commercial hub of Nigeria	=1 if Yes; 0 if No
Group	Binary measure of whether the firm is part of a group of companies	=1 if Yes; 0 if No
Export	Binary measure of whether the firm is an exporter	=1 if Yes; 0 if No

### Estimation method

We employ a simplified economic model to predict the likelihood of innovation outcomes in our sample. We assume that the probability of innovation is the function of formal coopetition and informal coopetition and absorptive capacity of an SME should moderate this relationship. Our model is presented in the specification I below:


$$Y_{it}=f(X_{it-1},\beta )\;(\text{I})$$

In our specification I, the dependent variable ( *Y _it_*) is either the probability of product innovation or the process innovation outcome of an SME “ *i*” in time “ *t*”. Further, ( *X*_*it*-1_) is a vector of independent variables (main explanatory and control variables) and “ *β”* is a vector of estimation parameters in the preceding year time ( *t-1*).

Given the binary nature of the dependent variables, a discrete choice model is the most appropriate to estimate their response to the explanatory variables. We estimate a bivariate probit equation to elicit the relationship between coopetition and innovation (product and process) in the Nigerian service sector.

The implementation of one type of innovation is associated with the likelihood of the other types ( Egbetokun, 2015). Consequently, if separate equations are estimated for each innovation type, the error terms from the independent equations are likely to be pairwise correlated, leading to biased and possibly inconsistent point estimates. This is a problem ignored in some studies such as the one by Carvalho *et al.* ( Carvalho *et al.*, 2013).

By estimating a simultaneous system of two equations and allowing the error terms to be freely correlated across equations, the bivariate probit makes it possible to obtain unbiased estimates when the dependent variables in a set of equations are potentially interdependent ( Freedman & Sekhon, 2010). A similar approach has been used extensively in previous studies ( *e.g.*, Egbetokun, 2015; Freitas *et al.*, 2011).

Table 3 reports the descriptive statistics and the correlation matrix. We check the issue of multicollinearity by computing the variance inflation factor (VIF) before and after the estimations. The mean VIF (1.21) was well below the acceptable threshold of 10 ( Neter *et al.*, 1985). These values indicate that the estimation data do not suffer from serious problems of multicollinearity.

**Table 3.** Descriptive statistics and correlation matrix.

*Variable*	*N*	*Min*	*Max*	*Mean*	*S.D.*	*1*	*2*	*3*	*4*	*5*	*6*	*7*	*8*	*9*	*10*
***Dependent variables***															
Product innovation	421	0	1	0.613	0.488										
Process innovation	421	0	1	0.694	0.462										
***Independent variables***															
1. Formal coopetition	421	0	1	0.14	0.348	1									
2. Informal coopetition	421	0	1	0.648	0.478	0.269*	1								
3. Staff quality	421	0	1	0.562	0.28	0.049	0.134*	1							
4. Other collaboration partners	421	0	1	0.945	0.288	0.007	-0.002	-0.061	1						
5. Staff training	421	0	1	0.648	0.478	0.183*	-0.448*	0.084	-0.046	1					
6. Size	421	2.197	8.854	3.653	1.297	0.019*	-0.059*	0.188*	-0.089	-0.059	1				
7. Age	421	0	4.852	2.326	0.878	0.083	0.077	0.026	-0.054	0.017	0.213*	1			
8. Location	421	0	1	0.62	0.486	0.062	0.089	0.192*	-0.124*	0.076	0.103*	0.04	1		
9. Group	421	0	1	0.238	0.426	0.016	0.119*	0.114*	0.036	0.096*	0.228*	0.084	-0.038	1	
10. Export	421	0	1	0.188	0.391	-0.089	0.099*	0.088	-0.125*	0.044	0.229*	0.172*	0.094*	0.171*	1
***Mean (max) VIF = 1.21 (1.59)***															

## 6. Results

### Descriptive results

We first examine the distribution of cases for our dependent variables as a function of the main independent variables. The results of this univariate analysis are presented in Table 4. Significant Z-test scores for both the product and process innovation variables suggest that we reject the null hypotheses 1a and 1b.

**Table 4.** Univariate analysis of the relationship between innovation and coopetition.

	Formal coopetition			Informal coopetition		
Rate of innovation	Yes	No	Comparison (z-tests)	Yes	No	Comparison (z-tests)
**Product (%)**	69.4	59.9	1.39 *	73.9	37.8	7.27 **
**Process (%)**	81.3	67.4	2.16 **	86.4	37.8	10.33 **

*Significant at 10% **significant at 5%

Table 5 details the share of innovative manufacturing and service firms that respectively engage in formal and informal coopetition. From the table we see that the share of service firms that innovated and engaged in formal coopetition is comparable to the share of manufacturing firms that did the same, across all innovation types. However, a significantly higher percentage of innovative service firms engaged in informal coopetition, compared to their manufacturing counterparts. This difference is most pronounced across process and marketing innovation where 11% more innovative service firms engaged in informal coopetition than in manufacturing.

**Table 5.** Industrial share of the sample according to coopetition types.

Innovation	Formal coopetition		Informal coopetition	
	Service	Manufacturing	Service	Manufacturing
Product	15.5	14.89	**78.29**	**70.42**
Process	16.1	14.09	**80.82**	**69.39**
Marketing	13.84	11.1	**69.18**	**57.45**
Organizational	13.77	12.16	**65.56**	**57.88**

Bold values are significantly different across rows at 5%.

Table 6 further demonstrates that a comparable share of service and manufacturing firms engaged in formal or informal collaboration with any type of actor. However, the share of service firms that collaborated with their competitors is 7% points more than the share of manufacturing firms. This difference is statistically significant at the 5% level.

**Table 6.** Industrial comparison of the sample.

	Service (n=469)	Manufacturing (n=890)
Formal cooperation	32.2	30.9
Informal cooperation	78.89	75.96
	Service (n=421)	Manufacturing (n=797)
Formal coopetition	13.78	11.92
Informal coopetition	**64.61**	**57.21**

Bold values are significantly different across rows at 5%.

In terms of absorptive capacity, Table 7 shows that service firms appear superior to the manufacturing firms. The average share of employees with a university degree is twice as high among the service firms compared to their manufacturing counterparts. We also found that service firms that engage in informal coopetition have significantly higher absorptive capacity (60% employees with university degree) compared to those that did not coopete informally (49% employees with university degree). This difference is not found among service firms that engage in formal coopetition nor among manufacturing firms in general.

**Table 7.** Industrial comparison of the sample.

Percent of graduate staff	Mean	SD
Service	0.56	0.28
Manufacturing	0.28	0.26

Taken together, the above presented results suggest that service firms show some substantial difference from their manufacturing counterparts in terms of coopetition, particularly of the informal type. The effects of coopetition in services, therefore, seems to merit a closer look.

### Estimation results

Table 8 reports the results for our bivariate probit model for product (Model 1) and process innovation (Model 2). The results reveal that *Formal coopetition* is negatively and significantly associated with the likelihood of product innovation ( *p<0.05*), whereas its effect on the probability of process innovation is statistically insignificant (see Model 1 and 2 of Table 8, respectively). Our hypothesis 1a predicts that formal coopetition is negatively associated with the probability of innovation. However, these results only support our hypothesis for product innovation (Model 1) and do not lend any support to our hypothesis for process innovation (Model 2). The effects of *Informal coopetition* are significant and positive for the probability of product innovation (p<0.01) in Model 1 and the probability of process innovation (p<0.01) in Model 2. We posited in our hypothesis 1b that informal coopetition is positively associated with the probability of innovation, and our results support this argument. The results further indicate that out of our two proxies for the absorptive capacity of a firm, *Staff quality,* has no statistically significant effect on the probability of product innovation, whereas it shows a significant negative effect on the probability of process innovation (p<0.1). Furthermore, *Staff training* shows significant positive effects on the probabilities of product innovation and process innovation.

**Table 8.** The relationship between coopetition and innovation in Nigerian service firms (bivariate logit estimations, 2005–2007; 2008–2010).

	(1)	(2)
	Product innovation	Process innovation
Formal coopetition	−1.234 ^**^	−0.881
	(0.626)	(0.711)
Informal coopetition	1.929 ^***^	1.999 ^***^
	(0.410)	(0.452)
Staff quality	0.397	−0.962 ^*^
	(0.491)	(0.523)
Staff training	1.281 ^***^	1.899 ^***^
	(0.263)	(0.277)
Formal coopetition*staff quality	1.059	−0.298
	(0.664)	(0.761)
Informal coopetition*staff quality	−1.593 ^***^	0.481
	(0.587)	(0.634)
Formal coopetition*staff training	0.394	1.067 ^*^
	(0.549)	(0.569)
Informal coopetition*staff training	−0.762 ^**^	−1.749 ^***^
	(0.351)	(0.403)
Other collaboration partners	−0.233	0.258
	(0.343)	(0.377)
Size	0.083	0.109
	(0.068)	(0.070)
Age	0.123	0.092
	(0.096)	(0.089)
Location	0.311 ^**^	−0.180
	(0.157)	(0.176)
Group	0.398 ^**^	0.265
	(0.177)	(0.189)
Export	0.055	0.006
	(0.216)	(0.230)
Year	−0.563 ^***^	−0.319
	(0.211)	(0.217)
Sector dummies	Yes	Yes
Observations	421	
Log likel.	−349.854	
Chi sq.	330.964 ^***^	
Rho	0.321 ^***^	

Standard errors in parentheses; ^*^ *p*<0.1, ^**^ *p*<0.05, ^***^ *p*<0.01

The coefficients of interaction variables in Models 1 and 2 depict the moderating effects of absorptive capacity ( *Staff quality* and *Staff training*) on *Formal coopetition* and *Informal coopetition* through the effects of interaction terms in relation to the likelihood of product and process innovation, respectively. The results in Model 1 reveal that the absorptive capacity of a firm, *when Staff quality* is considered *,* negatively moderates (p<0.01) the probability of product innovation through informal coopetition (Model 1).

Further, the coefficients of the interaction effects of *Staff training* demonstrate that the absorptive capacity of a firm positively moderates (p<0.1) the probability of process innovation through formal coopetition and negatively moderates both the probabilities of product (p<0.05) and process innovation (p<0.01) through informal coopetition. According to our hypotheses 2a and 2b, we expected a positive moderating effect of absorptive capacity on the association of formal coopetition and innovation (product and process), and a negative moderating effect on the association of informal coopetition and innovation. However, our hypothesis 2a holds only in case of *Staff training* for process innovation, and our hypothesis 2b holds except in the case of *Staff quality* for process innovation. In terms of control variables, the coefficients of *Location* and *Group* are positive and significant for product innovation, whereas, insignificant for process innovation.

## Discussion and conclusion

In this study, we analyze the role of formal and informal coopetition in the innovation activities of SMEs in Nigeria, as well as the moderating effect of the absorptive capacities on the prospects of coopetition. Our results indicate that informal coopetition has a significant and positive effect on innovation, both product and process innovations, whereas we find that formal coopetition effects only the output of product innovation. Studies in innovation cooperation endorse the similar findings ( *e.g.*, Bönte & Keilbach, 2005). Moreover, our findings also support the argument that absorptive capacity moderates the relationships between coopetition and innovation.

Our study contributes to the literature on coopetition in the following ways. First, the study enhances our understanding of the concept of coopetition in the context of an emerging economy, Nigeria. Although a number of studies have addressed the feature of coopetition in advanced economies ( Arranz & de Arroyabe, 2008; Nieto & Santamaría, 2007), little attention has been paid to the effect of coopetition on innovation in developing and emerging countries. Second, this study focused on a largely understudied aspect of coopetition: that is, the nature of interaction among firms in coopetition. This aspect becomes more relevant to the institutional and the cultural contexts of the emerging economies of Africa and Asia, where social capital plays an important role in business dynamics. Finally, we base our empirical analysis on data of service firms. This provides us with valuable insights on how the coopetition relates to the activities of service firms.

Building on existing debate about coopetition in management and innovation literature, this study increases our understanding of how formal and informal coopetition interplay among the “frenemies” of innovation in Nigeria. In this study, we argue that the formal coopetition is a risky process due to the risk of reciprocal knowledge transfer and double coincidence of wants, whereas informal coopetition can mitigate these risks and leads to innovation. Moreover, since the innovation is an interactive mechanism and coopetition is inherently risky, the internal capabilities of firms define their potential success in such collaboration, and therefore, absorptive capacity of a firm plays an important role in the interactions of innovations.

Despite its contributions, our study has some limitations. First, due to the data limitations and data collection issues in developing countries, we are not able to include market-specific contextual factors in our analysis. Second, the nature of our survey limits our methodological prospects (e.g., mostly binary variables). Finally, on the similar grounds, our dataset does not allow us to fully address the size-, ownership, and industry-specific heterogeneities. Along these lines, the future research should focus on including more contextual elements to research. It would be interesting to investigate deeply if context plays a role in managing coopetition relationships beyond the nature of interactions, and what would be some new and novel theoretical insights that could be generated in this regard. Moreover, since binary variables do not fully capture the potential of heterogeneity, future research should complement the data with other secondary information to have meaningful insights. Another important avenue for future research could be the comparison of the issue of coopetition and innovation in advanced economies and transforming economies. Similarly, although we have addressed the issue of endogeneity between coopetition and innovation to an extent in our analysis, future research should focus on richer analyses of more detailed data to isolate the role of coopetition in innovation activities of SMEs.

## Data availability

### Underlying data

Data are from the Nigerian innovation survey data, collected with NEPAD support, prepared with PEDL funding and are publicly available at https:/doi.org/10.17632/37pys4vxt4.1

Data are available under the terms of the Creative Commons Attribution 3.0 International license (CC-BY 3.0).

## Footnotes

^1^ Here the term “frenemies” is a contraction of two words: “friends” and “enemies”. The term reflects that the same actors could simultaneously be both friends (supporting) and enemies (inhibiting) of innovation in a coopetition scenario.
