# Supplementary material for: “Frenemies” of innovation: understanding the role of coopetition in service innovation in emerging markets
Source: Open Res Eur. 2022 Jun 21;2:25. Originally published 2022 Feb 24. [Version 2] doi: 10.12688/openreseurope.14472.2 (PMC10446029; doi:10.12688/openreseurope.14472.2)
Supplement: Peer review history

# Peer review: Reviewer response for version 2

**DOI:** 10.21956/openreseurope.16082.r29566

**Published:** 2022-07-08

**Authors:** Elvis Avenyo

**Affiliations:** 1 Centre for Competition, Regulation and Economic Development (CCRED), DSI/NRF South African Research Chair in Industrial Development, University of Johannesburg, Johannesburg, South Africa

Thanks to the authors for considering my earlier comments. I am satisfied with the attempt made to incorporate the comments despite their data constraints.

If applicable, is the statistical analysis and its interpretation appropriate?

Partly

Is the study design appropriate and is the work technically sound?

Yes

Is the work clearly and accurately presented and does it engage with the current literature?

Yes

Are the conclusions drawn adequately supported by the results?

Partly

Are sufficient details of methods and analysis provided to allow replication by others?

Yes

Are all the source data and materials underlying the results available?

No

Reviewer Expertise:

My related focus areas of research are on innovation in African firms, technological change, industrial policy, among

I confirm that I have read this submission and believe that I have an appropriate level of expertise to confirm that it is of an acceptable scientific standard.

# Peer review: Reviewer response for version 1

**DOI:** 10.21956/openreseurope.15625.r28648

**Published:** 2022-04-01

**Authors:** Elvis Avenyo

**Affiliations:** 1 Centre for Competition, Regulation and Economic Development (CCRED), DSI/NRF South African Research Chair in Industrial Development, University of Johannesburg, Johannesburg, South Africa

The article focuses on understanding the relationship between coopetition and the likelihood of innovation using data on 421 SMEs in Nigeria. The data is a repeated cross-section collected in 2008 and 2011.

 In general, the paper is well-written with very sound motivation and structure. The paper is also succinct and the authors seem well vested in the literature.

 However, there are major methodological gaps some of which I highlight below.

- While cooperation between competing firms is an interesting concept and may raise issues around collusion, this is not expected among SMEs given the size and market shares of SMEs. A question however is how the benefits of innovation are appropriated, and if this endogenously influences coopetition and innovation. 
- Also, the nature of the collaborations matter. Given that firms are competitors, one can assume that they are similar on several dimensions, and the nature of coopetition and innovation may differ across sectors and firm sizes.

If this holds, it may be interesting to know what drives this kind of heterogeneous cooperation in SMEs, and if smaller firms benefit from such coopetitions.

 Authors can conduct split sample analysis to observe some of these heterogeneities if they exist.

 These could be an even more interesting angle to the paper beyond the formal and informal coopetiton analysis.

- Hypotheses 1 should be rephrased as: ..... 'probability to innovate'
- The authors indicate that they use data on 469 SMEs across two periods. Good to indicate the distributions across the two periods.
- One can argue that the real effect of coopetition is not expected to be seen on the probability of innovation, but on how these innovations perform. Product innovation in markets given that these firms compete for market size once innovation occurs. Could this analysis be done for at least on product innovation given that this is mostly measured in CIS-like datasets?
- In emerging markets where there is high informality, it may be critical to examine the type of competitor, be it formal or informal SMEs. There is empirical literature that shows that informal and formal competitions generate different innovation outcomes in SMEs in developing countries.
- On page 7, the authors present and compare descriptive stats on both services and manufacturing SMEs. Could the authors explain the purpose for the comparison if the focus is on service firms?

If data exist on manufacturing firms, why is the analysis not covering both sectors, and possibly examining whether coopetition happens in service and manufacturing firms differently? 

 I am a bit unclear why the focus is on service firms. I would suggest the analysis is expanded to include manufacturing firms

- In the analysis, the authors introduced both types of coopetiton separately into the model; is it possible that competition (computed as whether you engage in formal and/or informal), in general, generates positive innovation outcomes even before this decomposition? Also, firms may engage in both formal and informal coopetiton in SMEs, particularly in emerging markets. It is important to consider the complementary effects of this in the analysis.
- In addition, the analysis makes little attempt to control for endogeneity between innovation and coopetition - firms may coopete to innovate or otherwise, and may also innovate in order to engage in beneficial coopetition. The results may be driven largely by this bidirectional relationship.

Minor:

 typo on page 6: 'whether' instead of 'weather'

If applicable, is the statistical analysis and its interpretation appropriate?

Partly

Is the study design appropriate and is the work technically sound?

Yes

Is the work clearly and accurately presented and does it engage with the current literature?

Yes

Are the conclusions drawn adequately supported by the results?

Partly

Are sufficient details of methods and analysis provided to allow replication by others?

Yes

Are all the source data and materials underlying the results available?

No

Reviewer Expertise:

My related focus areas of research are on innovation in African firms, technological change, industrial policy, among

I confirm that I have read this submission and believe that I have an appropriate level of expertise to confirm that it is of an acceptable scientific standard, however I have significant reservations, as outlined above.

# Response

**Published:** 2022-04-23

**Authors:** Sohaib S. Hassan

**Affiliations:** University of Siegen, Germany

Dear Reviewer, Thank you for your valueable feedback. We are working on addressing all your concerns. We'll submit a revised version with a detailed response soon. Thank you one again. Best Regards, Sohaib & Co-Authors

# Response

**Published:** 2022-06-13

**Authors:** Sohaib S. Hassan

**Affiliations:** University of Siegen, Germany

Dear Dr. Avenyo ,   Thank you very much for sparing time to review our manuscript. Indeed, your observations about our paper are quite relevant and we intend to address your concerns in the new version of our study.  We have carefully examined all your observations and prepared our response below and edited the manuscript where necessary. Should you want us to make additional changes, we would be happy to comply.   Thank you very much once again.   Yours sincerely,

 Sohaib & Co-Authors _______________________________________________________________ **Reviewers’ Comments and responses**   The article focuses on understanding the relationship between coopetition and the likelihood of innovation using data on 421 SMEs in Nigeria. The data is a repeated cross-section collected in 2008 and 2011.

 In general, the paper is well-written with very sound motivation and structure. The paper is also succinct and the authors seem well vested in the literature.

 However, there are major methodological gaps some of which I highlight below.

- While cooperation between competing firms is an interesting concept and may raise issues around collusion, this is not expected among SMEs given the size and market shares of SMEs. A question however is how the benefits of innovation are appropriated, and if this endogenously influences coopetition and innovation. 
- Also, the nature of the collaborations matter. Given that firms are competitors, one can assume that they are similar on several dimensions, and the nature of coopetition and innovation may differ across sectors and firm sizes.
- If this holds, it may be interesting to know what drives this kind of heterogeneous cooperation in SMEs, and if smaller firms benefit from such coopetitions.
  Authors can conduct split sample analysis to observe some of these heterogeneities if they exist.
  These could be an even more interesting angle to the paper beyond the formal and informal coopetiton analysis.

 

- One can argue that the real effect of coopetition is not expected to be seen on the probability of innovation, but on how these innovations perform. Product innovation in markets given that these firms compete for market size once innovation occurs. Could this analysis be done for at least on product innovation given that this is mostly measured in CIS-like datasets?

 

- In emerging markets where there is high informality, it may be critical to examine the type of competitor, be it formal or informal SMEs. There is empirical literature that shows that informal and formal competitions generate different innovation outcomes in SMEs in developing countries.

**Response:** Thank you very much for your comments. We agree with your comments and thus, we have provided the related debate in the manuscript. Please see below: The research about coopetition among organizations largely focuses on large organization, as SMEs tend to differ from large firms in terms of their competences and priorities. Further, due to their rather unsystematic structures, SMEs show relatively less interest in pursuing coopetition strategies to gain performance outcomes (Morris et al. 2007; Roig-Tierno et al. 2018). However, while SMEs are generally market oriented and tend to mainly focus on growth and developing their core businesses, they need to be innovative in competitive markets to overcome their liabilities of smallness (Bengtsson and Johansson, 2014; Roig-Tierno et al. 2018). Coopetition alliances might facilitate their competitive advantage, access to new markets and survival (Morris et al. 2007; Kraus et al. 2012). The existing research indeed has pointed to such practices among SMEs (e.g., Kraus et al. 2012; Bouncken et al. 2015; Devece et al. 2017; Crick and Crick, 2021; Gernsheimer et al. 2021), but the empirical evidence is rather limited in scope (sectoral and geographical). Our study addresses this limitation of the literature by providing empirical evidence pertaining to the role of coopetition in innovation among SMEs in a large developing country. We also underscore that in an emerging country settings SMEs may not collude, but they engage in coopetition. This is especially true in large developing countries like Nigeria where most of the firms are SMEs. Sometimes, the only way they manage to survive and compete in the potentially oligopolistic market is through partnerships. Your observation also alludes to the issue of appropriability and radical innovations which, while very relevant, is beyond the scope of our study and also limited by our data structure. As for the size specific heterogeneity and innovation types, your concerns are quite apt. However, we find no evidence of size heterogeneity in a preliminary analysis including an interaction term of the main explanatory variables with size. Thus, our results are not meaningful in this regard. We are convinced that this is because most of the firms are small and so will not necessarily vary based on size in the nature and benefits of coopetition. Besides, our small sample size does not allow us to examine such heterogeneities.   ______________________________________________________________  

- On page 7, the authors present and compare descriptive stats on both services and manufacturing SMEs. Could the authors explain the purpose for the comparison if the focus is on service firms?
- If data exist on manufacturing firms, why is the analysis not covering both sectors, and possibly examining whether coopetition happens in service and manufacturing firms differently? 

I am a bit unclear why the focus is on service firms. I would suggest the analysis is expanded to include manufacturing firms

- In the analysis, the authors introduced both types of coopetiton separately into the model; is it possible that competition (computed as whether you engage in formal and/or informal), in general, generates positive innovation outcomes even before this decomposition? Also, firms may engage in both formal and informal coopetiton in SMEs, particularly in emerging markets. It is important to consider the complementary effects of this in the analysis.
- In addition, the analysis makes little attempt to control for endogeneity between innovation and coopetition - firms may coopete to innovate or otherwise, and may also innovate in order to engage in beneficial coopetition. The results may be driven largely by this bidirectional relationship.

**Response:** We have noticed some typos in the ORE formatted version of our manuscript. Please excuse our oversight in this regard. We have corrected the tables to reflect the correct information. Overall, Since the focus of this research is on rather the underexplored industrial dimension (service sector) of coopetition in emerging markets, we had excluded the manufacturing sector from our main analysis. However, to demonstrate how there exist some differences between service and manufacturing sectors vis-à-vis the types of coopetition, we include this feature in our analysis. The information in Table 4 and Table 5 (corrected versions. See here: *https://s3-eu-west-1.amazonaws.com/openreseurope/linked/199849.Tables_4%2C5%2C6.docx* ) highlight some of the differences between the service and manufacturing sectors which, in our opinion, warranted our sole focus on the service sector. Generally, we find informal channel of coopetition to be significant for all types of innovation in our sample SMEs albeit the practice is statistically significantly more divergent among the service sectors SMEs. The tables are reproduced below. A significantly higher percentage of innovative service firms engaged in informal coopetition, compared to their manufacturing counterparts. This difference is most pronounced across process and marketing innovation where 11% more innovative service firms engaged in informal coopetition than in manufacturing. Thus, this justifies, to an extent, our focus on the service sector SMEs. We have estimated our model for the manufacturing SMEs. The results are presented in the Table A . Further, please see results for the combined effect in the Table B (See here for Tables A & B *https://s3-eu-west-1.amazonaws.com/openreseurope/linked/199850.Tables_A%2C_B.docx* ). It is indeed true that both formal and informal coopetition are often used together, and, in combination, they generate positive effects on both product and process innovation. However, the distinction we make between both types in our analyses allow us to uncover which type may have a stronger effect on innovation. The issue of endogeneity is a quite relevant one. Although we have aimed to reduce the issue of endogeneity with our econometric modelling, one cannot deny the technical aspect of it. However, contextually speaking, it is almost impossible in a business environment like Nigeria where innovation is very expensive due to institutional, infrastructural and credit deficits that a firm would innovate just because it wants to coopete. We have nonetheless included this limitation of our study in the last section of the manuscript to highlight the issue. ____________________________________________________________ **Minor Comments:**             Hypotheses 1 should be rephrased as: ..... 'probability to innovate' **Response: **We have edited it as advised.

 The authors indicate that they use data on 469 SMEs across two periods. Good to indicate the distributions across the two periods. **Response: **The information has been indicated in the sub-section: “Data and Sample” under the section: “Methods” typo on page 6: 'whether' instead of 'weather' **Response: **Corrected _______________________________________________________________   New References:

- Crick, J. M., & Crick, D. (2021). Coopetition and family-owned wine producers. Journal of Business Research, 135, 319-336.
- Gernsheimer, O., Kanbach, D. K., & Gast, J. (2021). Coopetition research-A systematic literature review on recent accomplishments and trajectories. Industrial Marketing Management, 96, 113-134.
- Roig-Tierno N, Kraus S, Cruz S (2018) The relation between coopetition and innovation/entrepreneurship. Rev Manag Sci 12(2):379–383
- Bengtsson M, Johansson M (2014) Managing coopetition to create opportunities for small firms. Int Small Bus J 32(4):401–427
- Kraus S, Pohjola M, Koponen A (2012) Innovation in family firms: an empirical analysis linking organizational and managerial innovation to corporate success. Rev Manag Sci 6(3):265–286
- Devece C, Ribeiro-Soriano DE, Palacios-Marqués D (2017) Coopetition as the new trend in inter-firm alliances: literature review and research patterns. Rev Manag Sci.
- Morris M, Koc ¸ak A, O ¨ zer A (2007) Coopetition as a small business strategy: implications for performance. J Small Bus Strategy 18(1):35–55
- Bouncken, R.B., Gast, J., Kraus, S. and Bogers, M. (2015), “Coopetition: a systematic review, synthesis, and future research directions”, Review of Managerial Science, Vol. 9 No. 3, pp. 577-601.

 

# Peer review: Reviewer response for version 1

**DOI:** 10.21956/openreseurope.15625.r28652

**Published:** 2022-02-25

**Authors:** Shoaib Abdul Basit

**Affiliations:** 1 Arden University Berlin, Berlin, Germany; 2 Faculty of Economics and Business Administration, Chemnitz University of Technology, Chemnitz, Germany

Very interesting research problem. The introduction section is well structured and clearly written in academic language. More recent literature needs to be cited in the theoretical framework to support your argument. As a general comment, note that although there are some references for 2014 or 2016, most are prior to 2012 and it would be useful to review and update the theoretical framework with recent literature.

 The study provides contributions to the current literature. The research method is well designed, and the appropriate analysis is performed. Further, the results and interpretations are written very well. The authors need to elaborate more on their findings in the discussion section and link them with the previous studies.

 Overall, the paper is well structured. I recommend this paper for indexing after making the minor changes.

If applicable, is the statistical analysis and its interpretation appropriate?

Yes

Is the study design appropriate and is the work technically sound?

Yes

Is the work clearly and accurately presented and does it engage with the current literature?

Yes

Are the conclusions drawn adequately supported by the results?

Yes

Are sufficient details of methods and analysis provided to allow replication by others?

Yes

Are all the source data and materials underlying the results available?

Yes

Reviewer Expertise:

Innovation and Management Strategies, Economics of Innovation, Entrepreneurship, Knowledge Sources, Open Innovation, Market Environment, Economic Development, International Trade and Public Policy, Sustainability Management

I confirm that I have read this submission and believe that I have an appropriate level of expertise to confirm that it is of an acceptable scientific standard.

# Response

**Published:** 2022-06-07

**Authors:** Sohaib S. Hassan

**Affiliations:** University of Siegen, Germany

Dear Dr. Abdul Basit, Thank you very much for your review. We have now revised our mansucript and updated the newer version on the platform. We have addressed your concern regarding the newer references. We have added newwer and more relevant references in our manuscript.   Thank you very much once again.   Best Regards, 

 Sohaib & Co-Authors
